# The Treatment of Intestinal Hemorrhage in Typhoid Fever

**Published:** 1893-10

**Authors:** 


### THE TREATMENT OF INTESTINAL HEMORRHAGE IN TYPHOID FEVER.

Dr. E. Maragliano, Professor of Clinical Medicine at the Medical Faculty of Genoa, maintains that the occurrence of intestinal hemorrhage during an attack of typhoid fever is not such a grave complication as is generally believed. Not only has he never lost a single patient from the acute anemia due to the accident—a statement which will probably be received with surprise by many a practitioner—but he is even of opinion that the occurrence of hemorrhage rather exerts a favorable influence on the subsequent progress of the disease. Indeed, the mortality among the cases under the Professor's care in which hemorrhage took place, was on the whole lower than among those which did not show this complication. This Prof. Maragliano brings forward as an argument in favor of his contention that bleeding is never injurious in infectious diseases, in spite of what may be said to the contrary, a certain quantity of the specific virus being thus removed from the organism.

In respect of the alleged influence of cold baths in increasing the liability to enterorrhagia in typhoid fever, Prof. Maragliano has found from experience that intestinal hemorrhage is of less frequent occurrence in cases of enteric fever which are treated by Brand's method than in others. Patients who suffer from hemorrhage are usually those in whom the treatment has been commenced too late, as, for example, those who are taken to the hospital when the disease is already far advanced.

The following is the method of treatment recommended by Prof. Maragliano in cases of enterorrhagia in the course of an attack of typhoid fever:

The physician should remain by the side of the patient until all signs of hemorrhage have disappeared. An ice-bag is to be applied to the abdomen especially over the right iliac foesa which is the seat of the hemorrhage. A hypodermic injection of one gramme (fifteen minims) of ergotine is at once to be made. From four to five grammes (sixty to seventy-five minims) of ergotine should be injected in the course of a few hours, for smaller doses will not produce the desired effect.

In addition to the above, one of the following cachets is also to be administered every hour:

℞. Powdered Opium...........gr. ij.
Subnitrate of Bismuth...... ʒ iij.
Mix and divide into twelve cachets.

Should the exaggerated peristaltic action of the intestine and the hemorrhage persist, the dose of opium is to be increased until twenty centigrammes (three grains) are taken in the twenty-four hours. Experience has shown that the action of full doses of opium is very well borne by typhoid patients.

Prof. Maragliano has succeeded in every case in arresting the hemorrhage by these means and sometimes even more rapidly than was to be expected.—*North American Practitioner.*

